# Study on the contamination of Abadan public parks soil with *Toxocara* spp. eggs

**DOI:** 10.1186/2052-336X-12-86

**Published:** 2014-05-19

**Authors:** Sharif Maraghi, Komeil Mazhab Jafari, Seyed Mahmoud Sadjjadi, Seyed Mahmoud Latifi, Mohammad Zibaei

**Affiliations:** 1 Department of Parasitology and Mycology, Abadan Arvand International division, Institute of Health, Thalassemia and Hemoglobinopathy Research Center, Ahwaz Jundishapur University of Medical Sciences, Ahwaz, Iran; 2 Abadan school of Medical sciences, Abadan, Iran; 3 Ahwaz Jundishapur University of Medical Sciences, Ahwaz, Iran; 4 Department of Parasitology and Mycology, School of Medicine, Shiraz University of Medical Sciences, Shiraz, Iran; 5 Department of Biostatistic and Epidemiology, School of Health, Health research institute, Diabetes research Center, Ahwaz Jundishapur University of Medical Sciences, Ahwaz, Iran; 6 Department of Parasitology and Mycology, School of Medicine, Alborz University of Medical Sciences, Karaj, Iran

**Keywords:** Toxocaraiasis, *Toxocara*, Contamination, Park, Soil, Abadan, Iran

## Abstract

**Background:**

Toxocariasis is one of the most important zoonotic diseases caused by *Toxocara* larva stage in humans. One of the major transmission routes of infection, especially in children is pica. The aim of this topic was study the contamination of Abadan public parks with *Toxocara* eggs.

**Materials and methods:**

Two hundred and ninety one samples of soil were collected from 31 parks. The samples were examined for *Toxocara* spp. eggs by modified floatation method using saturated sucrose. The results were analyzed using SPSS version 19 and Chi-square test.

**Results:**

Eighty five (29.2%) out of 291 samples were infected with *Toxocara *spp. eggs, means19 (61.2%) of the 31 parks were contaminated. There was no significant difference between the urban and suburb parks contamination (p = 0.208) but there was significant relation between contamination with *Toxocara *spp. eggs and traces of cats and dogs presence in the parks (p = 0.001).

**Conclusion:**

As the contamination of Abadan public parks soil with *Toxocara *spp. eggs is relatively high, the people and specially children might get the contamination during stay in the parks and measures should be taken to control the stray cats and dogs.

## Background

Toxocariasis is a zoonotic disease caused by *Toxocaracanis* and *Toxocaracati* Larvae in humans. Infection acquire via the ingestion of infective ova with vegetables or pica or by ingesting products of contaminated paratenic hosts [1-3]. The larva hatch in small intestine and immigrates to other organs, almost to liver and cause visceral larva migrans (VLM) or localizes in eyes and causes ocular larva migrans (OLM) [4-7].

Although dogs and cats are definitive hosts, but the expelled eggs should remain in the soil until larva develops within 6 week [5,6]. The infection of dogs and cats in Iran was proved by Epidemiological surveys [8-11].

Examination of Urmia public parks soil indicated that 7.8% of the parks were contaminated with *Toxocara* eggs [12], In Khoramabad 22.2% [13], In Tehran 38.7% [14], and in Shiraz 6.3% [8].

The aim of this topic was study the contamination of Abadan public parks soil with *Toxocara* eggs.

## Materials and methods

From January to April 2012, 291 soil samples were collected from 31 Abadan public parks of urban and suburb of Abadan southwest Iran. Each sample was 100 grams of soil with 3 cm ground depth. The samples were examined for *Toxocara* spp. using Zibaei et al. method [15]. We modified the method by replacement of sieve with mesh material and using saturated sucrose (1.2 g/cm3). In this method, each soil sample was grinded and dissolved in distilled water, mixed well and filtered by 3 layers of mesh material, then centrifuged in 2000 rpm for 5 minutes and supernatant was discarded and precipitation was re-suspended in normal saline. Then it was centrifuged again and the supernatant was discarded and precipitation was removed by shaking the tube and saturated sucrose was added and centrifuged in 1500 rpm for 15 minutes and then sucrose was added to filling the top of the tube. Then the coverslip was placed on the tube in touch with the sucrose and was kept in the rack for 45 minutes, and then the coverslip was removed and placed on a glass slide and studied under the light microscope.

## Results

**Figure 1:**
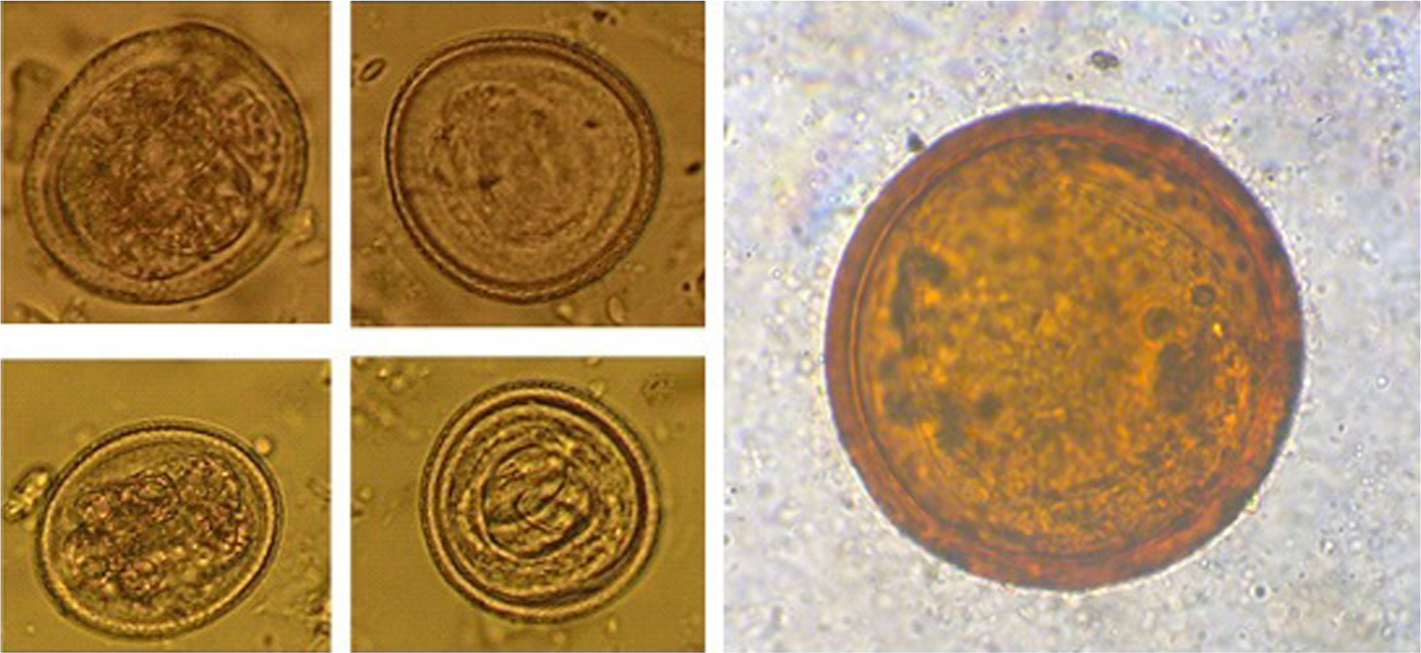
**Eggs of *Toxocara *** **Species isolated from the soil of Abadan parks (400×).**

Eighty five (29.2%) out of 291 samples were positive for *Toxocara* spp. eggs (Figure 1). The eggs were isolated from 19 (61.2%) parks. 23.7% of contaminations were in urban parks and 5.5% in suburban.

There was no significant difference between the contamination in urban and suburb (p = 0.208), but the contamination in parks where the pets were present was higher (p = 0.0001).

## Discussion

Soil transmitted and zoonotic helminthes are still one of the most important health problems in the world, ever in developing countries [16].

In this study the rate of soil contamination with *Toxocara* spp. egg in Abadan in south west Iran was 29.2%. The eggs were isolated from 61.2% of the parks.

In this study the sieve was replaced by application of mesh material which caused the water saving and accelerating the method and more eggs could be achieved.

The contamination in our study was lower than other reports such as Greece (97.5%), Germany (89.1%), Japan (63.3%), Brazil (60%), Malaysia (54. 5%), and Cuba (42.2%) [17-22].

The rate of contamination was higher than reports from USA (20.6%), Turkey (18.9), Iraq (15.5), London (6.3%) [23-26], Shiraz 6.3% [8], Ireland 5.6% [27], Urmia 3.9% [12], and Spain 1.2% [28].

The difference of contamination rate depends on culture, climates, methodology of examination and sample collection. Recent reports by Khazan et al. [29] and Tavalla et al. [14] indicated that the rate of Tehran soil contamination was 10% and 38.7% respectively; surprisingly the samples of both studies were collected in 2008.

Serological surveys with ELISA and Western blot techniques in hypereosinophil individuals in ahwaz indicated that 19% of them were positive for toxocariasis [30].Talaizadehet all reported 3 cases of toxocariasis in pathology and serological examination [31].

As the rate of infection with *Toxocara* species in dogs is varied from 10 to 51.6% [32,33] and in cats from 13 to 52.7% [10,34] and the pets have access to the parks, the rate of soil contamination in many parks in Iran is high [13,14].

## Conclusion

The result of this study indicated that the rate of soil contamination of Abadan public parks with *Toxocara*s pp. eggs is relatively high and measures should be taken to control the presence ofstray dogs and cats in Abadan.

## Competing interests

The authors declare that they have no competing interests.

## Authors’ contributions

KMJ collected the samples. SM, SMS and MZ carried out the examination of the samples. SML carried out the analysis of the data. All authors read and approved the final manuscript SM, KMJ, SMS, SML, MZ.
